# Synthesis and Aggregation Behavior of Temperature- and pH-Responsive Glycopolymers as Sugar-Displaying Conjugates

**DOI:** 10.3390/polym12040956

**Published:** 2020-04-20

**Authors:** Sotaro Tsuji, Tomohiro Aoki, Shunsuke Ushio, Tomonari Tanaka

**Affiliations:** Department of Biobased Materials Science, Graduate School of Science and Technology, Kyoto Institute of Technology, Kyoto 606-8585, Japan; m8661007@edu.kit.ac.jp (S.T.); m7661001@edu.kit.ac.jp (T.A.); m9661002@edu.kit.ac.jp (S.U.)

**Keywords:** glycopolymer, stimuli-responsive, random copolymer, *N*-isopropylacrylamide, methacrylic acid, aggregation

## Abstract

Stimuli-responsive polymers have attracted significant interest in the fields of advanced materials and biomaterials. Herein, temperature- and pH-responsive glycopolymers, which are composed of *N*-isopropylacrylamide, methacrylic acid, and an acrylamide derivative bearing a lactose moiety, were synthesized via radical copolymerization. The series of resulting glycopolymers had different degrees of substitution of the lactose moieties, were responsive to temperatures between 26.6 °C and 47.6 °C, and formed aggregates above the lower critical solution temperature limit in mild acidic aqueous media (pH 4–6). The temperature-responsive behavior was dependent on the prevailing pH conditions, as no aggregation was observed in neutral and basic aqueous media (pH > 7). The aggregates had saccharide moieties on the surface in aqueous media. The number of saccharide moieties on the surface depended on the saccharide-containing unit ratio in the glycopolymer. The ratio was determined via enzymatic hydrolysis of the lactose moieties using β-galactosidase and the subsequent detection of the released galactose.

## 1. Introduction

Stimuli-responsive polymers, such as temperature-, pH-, ion-, photo-, and magnetic field-responsive polymers, have recently attracted significant interest for their use as films, gels, and vesicles for advanced materials and biomaterial applications [1,2,3,4,5]. As the most well-known temperature-responsive polymer, poly(*N*-isopropylacrylamide) (PNIPAM) has a lower critical solution temperature (LCST) value of approximately 32 °C [6,7] which makes it water-soluble when the surrounding temperature is below the LCST. On the other hand, PNIPAM aqueous solutions become opaque when the surrounding temperature is above the LCST. This is because dehydration and aggregation processes prevail under these conditions. pH-responsive polymers, such as carboxylic acid group-containing polymers, are popular stimuli-responsive polymers that are of interest to researchers as they are particularly useful for biomedical applications.

Multi-stimuli-responsive polymers, such as temperature-, pH-, and/or photoresponsive polymers, are used in biomaterials and drug delivery systems [8,9,10,11,12]. Copolymers containing *N*-isopropylacrylamide (NIPAM) and methacrylic acid (MAA) are both temperature- and pH-responsive polymers, and their inherent properties have been extensively researched [9,13,14]. It is important to consider the conditions, including temperature and pH for the development of biomaterials and their applications. For example, some living systems such as early endosomes and the surface of the skin are working under mildly acidic conditions [15,16]. Glycopolymers, which are biofunctional synthetic polymers with pendant saccharides, are stimuli-responsive polymers. They are recognized by glycoreceptors such as lectins, viruses, and toxins [17,18,19,20]. Several reports have detailed the synthesis of multi-stimuli-responsive glycopolymers [21,22,23,24]. One report focused on the inherent properties of triple-stimuli-responsive glycopolymers. Here, Tang and Pei et al. reported about an amphiphilic glycopolymer that was temperature-, pH-, and light-responsive [23]. The versatility across many fields has necessitated the development of various types of multi-stimuli-responsive glycopolymers.

We recently reported on the use of a simple protocol for synthesizing glycopolymers from free saccharides without the need for a protecting group [25,26,27]. The protocol featured a direct anomeric activation reaction using a dehydrating condensing agent (often referred to as the Shoda activation) [28,29] and an azide–alkyne cycloaddition reaction (commonly referred to as click chemistry) [30]. It was used for the synthesis of the appropriate glycomonomer, followed by radical polymerization of the abovementioned glycomonomer to obtain the desired glycopolymer. The present protecting-group-free method for glycopolymers can be applied to mono-, di-, and oligosaccharides with a higher molecular weight, such as complex-type sialyloligosaccharides. In this paper, the synthesis of multi-stimuli-responsive glycopolymers was reported; particular interest was given to temperature- and pH-responsive and enzyme-recognized polymers. The glycopolymers composed of NIPAM, MAA, and a lactose (Lac)-bearing acrylamide derivative (LacAAm) that had been made using the abovementioned protecting-group-free method from a free saccharide. They were synthesized using controlled radical copolymerization. The aggregation behavior of these glycopolymers in various temperature and pH conditions were investigated in aqueous media. Moreover, the presence of the saccharide moieties on the surface of the aggregates was confirmed.

## 2. Materials and Methods

### 2.1. Materials

*N*-Isopropylacrylamide (NIPAM), methacrylic acid (MAA), and 2,2’-azobis(4-methoxy-2,4-dimethylvaleronitrile) (V-70) were purchased from FUJIFILM Wako Pure Chemical Corporation (Osaka, Japan). NIPAM was used after recrystallization from *n*-hexane. 2-(Benzylsulfanylthiocarbonylsulfanyl) ethanol (BTSE) [31] and the Lac-bearing acrylamide derivative (LacAAm) [26] were synthesized according to previously published methods. β-Galactosidase from *Aspergillus oryzae* and the galactose assay kit were purchased from Sigma-Aldrich Co., LLC (MO, USA). All other reagents were commercially available and used without further purification.

### 2.2. Measurements

NMR spectra were recorded using a Bruker (MA, USA) BioSpin AV-300 spectrometer. Gel permeation chromatography (GPC) measurements were conducted using a system consisting of a JASCO (Tokyo, Japan) PU-2089 pump, a JASCO CO-2065 column oven, a JASCO RI-2031 refractive index detector, and a Shodex OHpak SB-804 HQ column (8.0 × 300 mm, SHOWA DENKO, Tokyo, Japan). A phosphate buffer (20 mM, pH 7.0) containing 20% *N*,*N*-dimethylformamide was used as the eluent at a flow rate of 0.5 mL/min at 30 °C. Pullulan samples were used as standards. Transmittance was recorded using a Hitachi (Tokyo, Japan) U-2000 spectrometer. Dynamic light scattering (DLS) analyses were conducted using an Otsuka Electronics (Osaka, Japan) ELSZ-1000 at 667 nm. The samples for transmittance and DLS analyses were prepared using 0.5 wt % in aqueous media and filtered through a membrane filter (0.45 μm). The fluorescence intensity was recorded using a JASCO FP-6500 spectrometer.

### 2.3. Synthesis of Glycopolymers

The radical copolymerization reaction was performed using a reversible addition-fragmentation chain transfer (RAFT) polymerization protocol. Briefly, the initiator V-70, the chain transfer agent BTSE, and the monomers NIPAM, MAA, and LacAAm at total monomer concentration of 1.0 M were dissolved in dimethyl sulfoxide (DMSO) in a glass tube. The molar ratio of the monomer/BTSE/V-70 mixture was 150/1/0.5. The resulting solution was degassed via three freeze-thaw cycles before being sealed under vacuum in the glass tube and heated at 37 °C for 24 h. The products were purified by dialysis (Spectra/Por7 molecular weight cut-off (MWCO) = 3500, Spectrum Labs, CA, USA) against deionized water and freeze-dried to obtain the desired glycopolymers. ^1^H NMR was used to calculate the molecular weights of glycopolymers based on the terminal phenyl group proton signal.

^1^H NMR (300 MHz, D_2_O): δ (ppm) 8.1–8.0 (triazole), 8.1–7.2 (COOH, NHCO), 7.1–7.0 (phenyl), 5.7–5.6 (H-1 of Lac), 4.4–4.2 (H-1’ of Lac and NH–CH_2_–triazole), 3.9–3.4 (Lac and CH of NIPAM), 2.1–1.7 ((–CH_2_–CH–)_n_), 1.7–1.1 ((–CH_2_–CH–)_n_), 1.1–0.6 (CH_3_ of NIPAM and MAA).

### 2.4. Transmittance Analysis

A copolymer aqueous solution (0.5 wt %) was measured in a quartz cell (cell length = 10 mm) at 500 nm with stepwise temperature variations. The phosphate buffer (100 mM, pH = 6, 7, and 8) and the phosphate-citrate buffer (100 mM, pH = 4 and 5) were used as the aqueous media.

### 2.5. Detection of Saccharide on the Surface of Aggregates

The copolymer and β-galactosidase solutions in a phosphate-citrate buffer (100 mM, pH 5) were pre-incubated separately at 40 °C. The mixture of the polymer (1.0 mg) and β-galactosidase (16 U) in the buffer (50 μL) was incubated at 40 °C for 16 h. After removal of both the polymer and the enzyme via centrifugation (Amicon Ultracentrifuge filter, nominal molecular weight limit (NMWL) = 3 kDa, Merck, Darmstadt, Germany), the fluorescence intensity of the filtrate was measured using a galactose assay kit and a fluorescence spectrophotometer (λ_ex_ = 535 nm and λ_em_ = 587 nm).

## 3. Results and Discussion

### 3.1. Synthesis of Glycopolymers

**Scheme 1:**
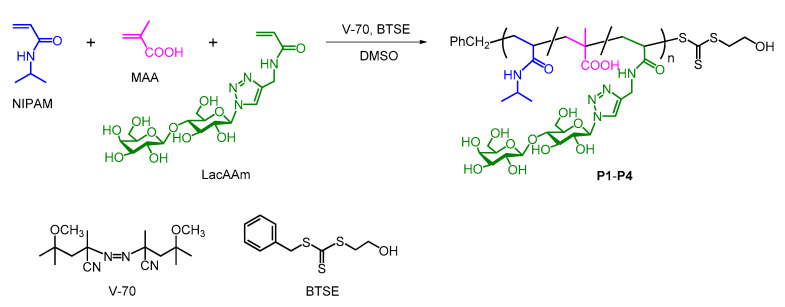
Synthesis of glycopolymers.

**Figure 1:**
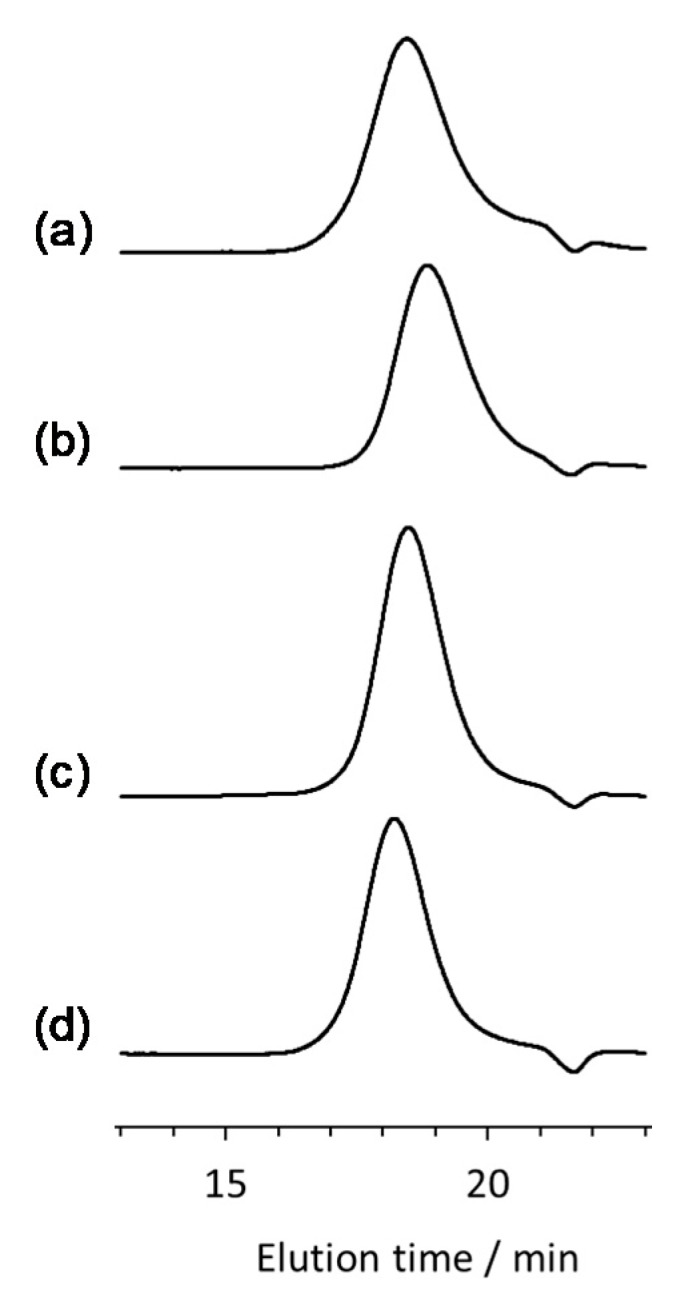
GPC chromatograms of (**a**) **P1**, (**b**) **P2**, (**c**) **P3**, and (**d**) **P4**.

**Figure 2:**
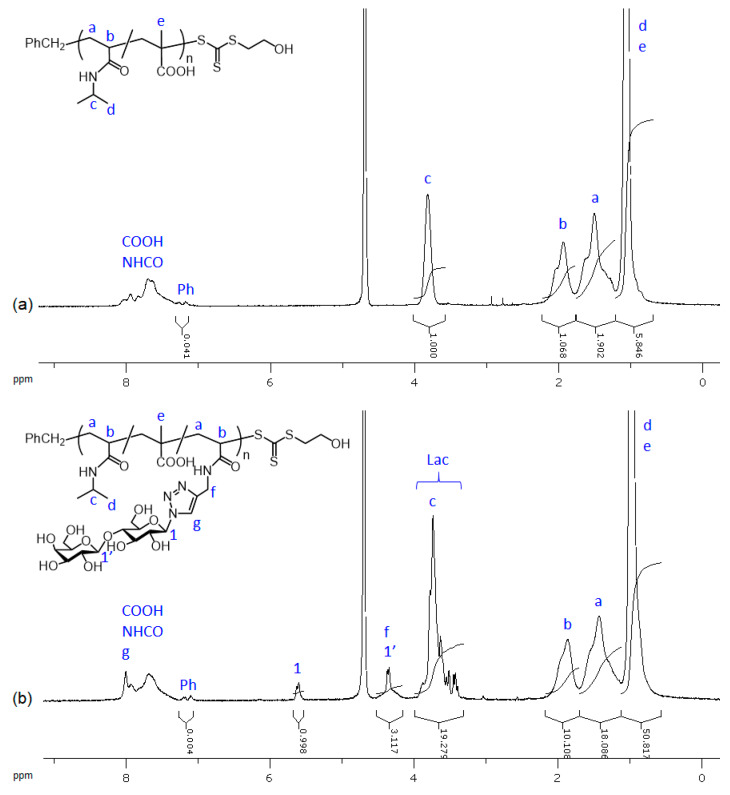
^1^H NMR spectra of (**a**) **P1** and (**b**) **P4** in D_2_O.

The synthetic route for the glycopolymers composed of NIPAM, MAA, and LacAAm is shown in Scheme 1. Here, the monomers were subjected to the RAFT copolymerization in DMSO to obtain the copolymers **P1**–**P4** using V-70 and BTSE as the initiator and the chain transfer agent, respectively. Performing the RAFT copolymerization at various monomer feeding molar ratios provided the desired glycopolymers with different ratios of LacAAm unit in their structures (Table 1). The ratio of the LacAAm unit in the product polymers agreed (0%–8.9%) with the feed ratio of LacAAm (0%–10%) as analyzed by ^1^H NMR. The GPC chromatograms obtained for **P1**–**P4** were monomodal with relatively narrow dispersity (*M*_w_/*M*_n_ = 1.27–1.33) (Figure 1). The ^1^H NMR spectra of the glycopolymer contained signals attributable to each monomer unit (Figure 2 and Figures S1,S2). The proton signals of the polymer backbone, the methyl protons of NIPAM and MAA, and the methine protons of NIPAM were observed at 2.1–1.1, 1.1–0.6, and 3.9–3.7 ppm, respectively. The proton signals of the Lac moiety were seen at 5.7–3.4 ppm. These results indicated that the glycopolymers composed of NIPAM, MAA, and LacAAm were synthesized successfully via the RAFT method.

### 3.2. Aggregation Behavior of Glycopolymers in Aqueous Media

**Figure 3:**
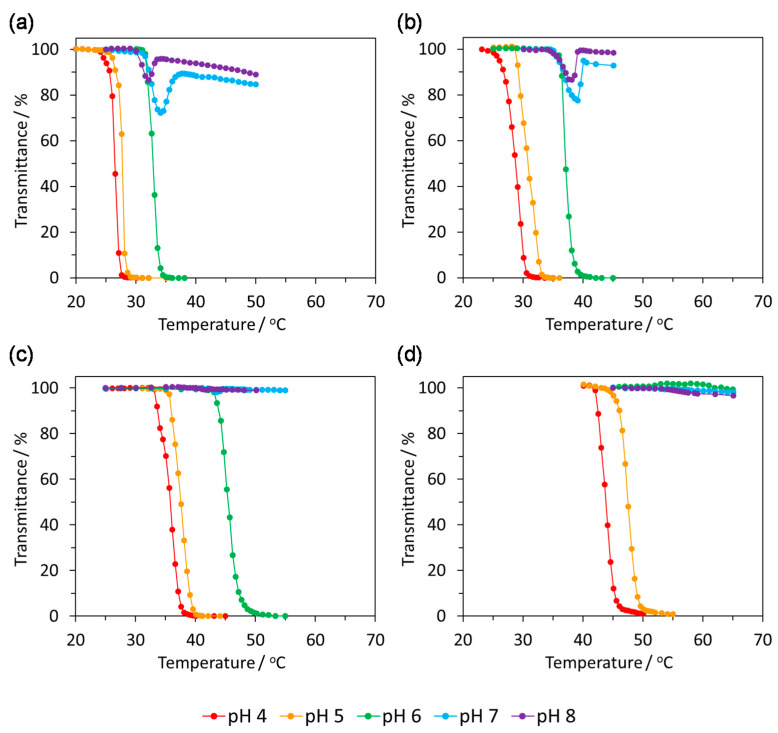
Transmittance of copolymer aqueous solutions (**a**) **P1**, (**b**) **P2**, (**c**) **P3**, and (**d**) **P4**. Here, red is pH 4, yellow is pH 5, green is pH 6, blue is pH 7, and violet is pH 8.

The aggregation behavior of copolymers **P1**–**P4** was investigated in aqueous media. Figure 3 shows the transmittance of **P1**–**P4** aqueous solutions with various pH buffers (pH = 4, 5, 6, 7, and 8). The transmittance of **P1**, which had no LacAAm unit, in pH 6 buffer decreased at around 32 °C when the temperature was increased with stepwise. When the temperature was below the LCST, i.e., below 32.8 °C, **P1** was soluble in aqueous media, but the solution became opaque when the temperature was above the LCST. The copolymer solution changed from clear to cloudy with increasing temperature because PNIPAM dehydration had occurred above the LCST. This phenomenon was evidence of the occurrence of aggregates composed of a hydrophobic PNIPAM. When the pH was low (pH = 4 and 5), the LCST of **P1** decreased from 32.8 °C to 26.6 °C and 27.6 °C for pH 4 and pH 5, respectively. Meanwhile, no LCST was observed in the higher pH ranges (pH = 7 and 8). The LCST values of the copolymers are summarized in Table 2. The LCST values of the copolymers in aqueous media gradually increased with an increase in the unit ratio of LacAAm and higher pH values (from 4 to 6). When the unit ratio of LacAAm was at its highest at 8.9% in **P4**, the LCST value was 47.6 °C in a pH 5 buffer solution. However, no LCST was obtained for **P1**–**P4** in the pH 7 and 8 buffer solutions, whereas the temperature was notably higher. It is known that the addition of a hydrophilic unit, such as a saccharide-containing unit, to the PNIPAM-based copolymer, increases the LCST of the copolymer [32,33]. For example, Ehe et al. reported that the LCST values of PNIPAM bearing glucose (Glc) moieties increased significantly from 56 °C to 81 °C with an increase in the unit ratio of the Glc-bearing monomer from 9% to 15%. When the ratio of Glc-bearing monomer increased to 26%, no LCST was observed. This difference in the aggregation behavior of the copolymers in various pH aqueous media was caused by the presence of the MAA unit, which facilitated protonation of the carboxylic acid moiety in the copolymers. Thus, the deprotonation of MAA in the copolymer avoided the issue of aggregation.

**Figure 4:**
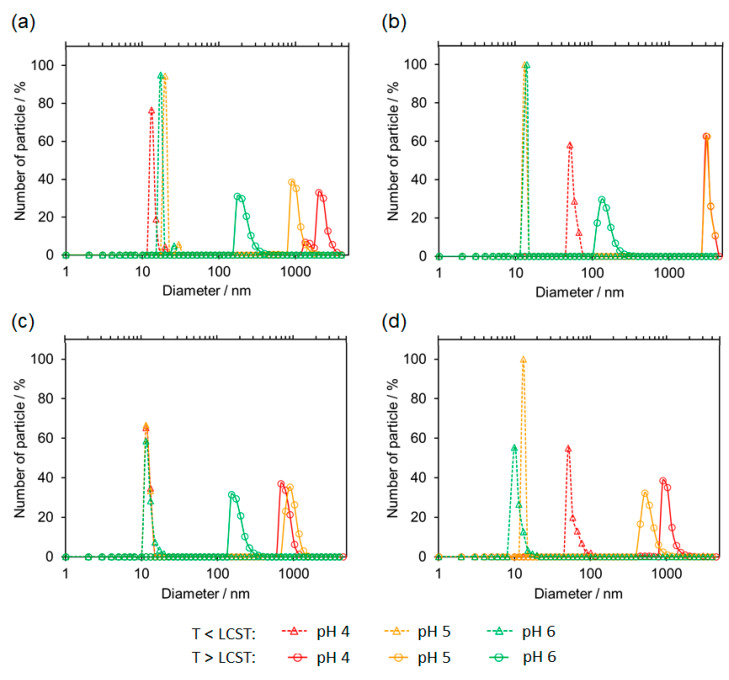
Dynamic light scattering (DLS) analyses of (**a**) **P1**, (**b**) **P2**, (**c**) **P3**, and (**d**) **P4** in different pH aqueous media. Here, red represents pH 4, yellow is pH 5, and green is pH 6. The triangles and broken lines represent below the LCST (**P1**: pH 4, 20 °C; pH 5, 20 °C; and pH 6, 25 °C. **P2**: pH 4, 20 °C; pH 5, 20 °C; and pH 6, 25 °C. **P3**: pH 4, 25 °C; pH 5, 25 °C; and pH 6, 25 °C. **P4**: pH 4, 25 °C; pH 5, 25 °C; and pH 6, 25 °C). The circles and solid lines represent above the LCST (**P1**: pH 4, 32 °C; pH 5, 32 °C; and pH 6, 38 °C. **P2**: pH 4, 32 °C; pH 5, 35 °C; and pH 6, 44 °C. **P3**: pH 4, 44 °C; pH 5, 44 °C; and pH 6, 53 °C. **P4**: pH 4, 50 °C and pH 5, 56 °C).

The diameter of the copolymer aggregates in aqueous media was analyzed by DLS (Figure 4). Here, we found that when the temperature of the copolymer solution was higher than the LCST value, the average diameter of the aggregates was bigger than the diameter seen below the LCST. In pH 4 and 5, the diameters were just over 1000 nm above the LCST. Meanwhile, no aggregation was observed when the temperature of the copolymer solution was lower than the LCST. Although the diameters of **P2** and **P4** in pH 4 analyzed by DLS were around 50 nm, the solutions were clear, and the copolymers were soluble in the buffer. It is suggested that small intermolecular interaction by hydrogen-bond between the amide group of PNIPAM and the carboxyl group of poly(methacrylic acid) (PMAA) might occur in case of pH 4 [14]. These results indicated that the aggregation behavior of the copolymers was temperature- and pH-dependent in mildly acidic aqueous media at the LCST boundary.

### 3.3. Detection of Saccharide on the Surface of the Aggregates

**Figure 5:**
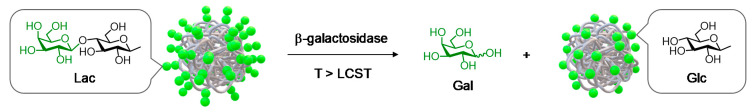
Schematic of the enzymatic treatment to confirm and quantify the saccharide moieties on the aggregates’ surface.

**Figure 6:**
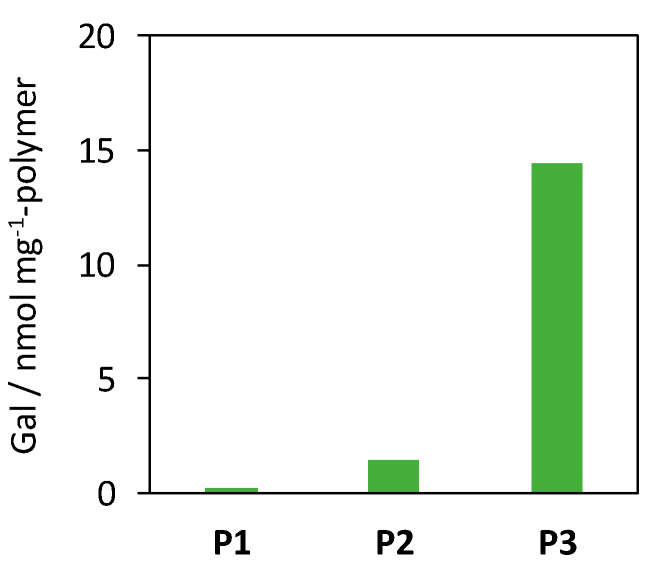
Released galactose from the aggregates in a pH 5 buffer solution above the LCST (40 °C).

The presence of hydrophilic saccharide moieties on the surface of the aggregates in aqueous media was confirmed via enzymatic treatment with β-galactosidase. Here, galactose (Gal) was released by enzymatic hydrolysis of the Lac moieties on the surface of the aggregates (Figure 5). The enzymatic hydrolysis by β-galactosidase was performed at 40 °C (above the LCST) in the pH 5 buffer solution since this was the optimal operating pH for the β-galactosidase. The released Gal was measured using a galactose assay kit and a fluorescence spectrophotometer. The amount of Gal released from **P3** (5.0% LacAAm) was higher than that released from **P1** and **P2** (0% and 1.8% LacAAm, respectively) (Figure 6). This result indicated that the aggregates in aqueous media above the LCST had saccharide moieties on the surface. The amount of saccharide on the surface of the aggregates increased when the molar ratio of the saccharide-containing unit in glycopolymers was high. The suggestion was that all Lac moieties were not displaying on the surface of the aggregates. In the case of the glycopolymer **P2**, which has lower LacAAm unit ratio, many Lac moieties are hidden in the aggregates. On the other hand, it is easy to display the Lac moieties of **P3**, which has higher LacAAm unit ratio, on the surface in aqueous media because of the higher hydrophilicity.

## 4. Conclusions

Temperature- and pH-responsive glycopolymers composed of NIPAM, MAA, and LacAAm were synthesized using a RAFT polymerization protocol. The LCST values of the resulting glycopolymers increased from 26.6 °C to 47.6 °C, with an increase in the unit ratio of LacAAm and higher pH values. Additionally, aggregation was observed above the LCST in aqueous media. These aggregates were composed of a dehydrated hydrophobic PNIPAM core and possessed hydrophilic saccharide moieties on their surface in aqueous media. In addition to contributing to the development of various types of biomaterials and drug delivery systems, it is believed that these multi-stimuli-responsive glycopolymers will promote more extensive research on other types of multi-stimuli-responsive glycopolymers.

## Figures and Tables

**Table 1:** Synthesis of glycopolymers.

Polymer	Feeding Molar Ratio of NIPAM/MAA/LacAAm	Conv. (%) ^a^	Yield (%) ^b^	*M*_n_ (g mol^−1^) ^a^	*M*_w_/*M*_n_^c^	Unit Ratio of NIPAM/MAA/LacAAm in Polymer ^a^
**P1**	95.0/5.0/0	75	76	14,300	1.27	94.9/5.1/0
**P2**	93.1/4.9/2.0	86	76	13,400	1.30	93.6/4.6/1.8
**P3**	90.2/4.8/5.0	90	72	16,600	1.33	90.5/4.5/5.0
**P4**	85.5/4.5/10.0	85	50	17,300	1.32	87.1/4.0/8.9

^a^ Determined by ^1^H NMR. ^b^ Isolated yield. ^c^ Determined by GPC.

**Table 2:** Lower critical solution temperature (LCST) values (°C) of copolymers.

Polymer	pH 4	pH 5	pH 6	pH 7	pH 8
**P1**	26.6	27.6	32.8	ND ^a^	ND
**P2**	28.6	30.1	37.1	ND	ND
**P3**	35.4	38.1	45.7	ND	ND
**P4**	43.9	47.6	ND	ND	ND

^a^ Not detected.

## Supplementary Materials

The following are available online at https://www.mdpi.com/2073-4360/12/4/956/s1, Figures S1 and S2: 1H NMR spectra of **P2** and **P3** in D_2_O.

## References

[1] De Las Heras Alarcón C., Pennadam S., Alexander C. (2005). Stimuli responsive polymers for biomedical applications. Chem. Soc. Rev..

[2] Ahn S.K., Kasi R.M., Kim S.C., Sharma N., Zhou Y. (2008). Stimuli-responsive polymer gels. Soft Matter.

[3] Li M.H., Keller P. (2009). Stimuli-responsive polymer vesicles. Soft Matter.

[4] Zhai L. (2013). Stimuli-responsive polymer films. Chem. Soc. Rev..

[5] Moad G. (2017). RAFT polymerization to form stimuli-responsive polymers. Polym. Chem..

[6] Roy D., Brooks W.L.A., Sumerlin B.S. (2013). New directions in thermoresponsive polymers. Chem. Soc. Rev..

[7] Lanzalaco S., Armelin E. (2017). Poly(*N*-isopropylacrylamide) and Copolymers: A Review on Recent Progresses in Biomedical Applications. Gels.

[8] Yuk H., Cho S.H., Lee S.H. (1997). pH/Temperature-Responsive Polymer Composed of Poly((*N*,*N*-dimethylamino)ethyl methacrylate-*co*-ethylacrylamide). Macromolecules.

[9] Tian P., Wu Q., Lian K. (2008). Preparation of temperature- and pH-sensitive, stimuli-responsive poly(*N*-isopropylacrylamide-*co*-methacrylic acid) nanoparticles. J. Appl. Polym. Sci..

[10] Chikh Alard I., Soubhye J., Berger G., Gelbcke M., Spassov S., Amighi K., Goole J., Meyer F. (2017). Triple-stimuli responsive polymers with fine tuneable magnetic responses. Polym. Chem..

[11] Qiao X.G., Dugas P.Y., Charleux B., Lansalot M., Bourgeat-Lami E. (2017). Nitroxide-mediated polymerization-induced self-assembly of amphiphilic block copolymers with a pH/temperature dual sensitive stabilizer block. Polym. Chem..

[12] Brooks W.L.A., Vancoillie G., Kabb C.P., Hoogenboom R., Sumerlin B.S. (2017). Triple responsive block copolymers combining pH-responsive, thermoresponsive, and glucose-responsive behaviors. J. Polym. Sci. Part A Polym. Chem..

[13] Brazel C.S., Peppas N.A. (1995). Synthesis and Characterization of Thermo-and Chemomechanically Responsive PolyCZV-isopropylacrylamide-co-methacrylic acid) Hydrogels. Macromolecules.

[14] Zhang J., Peppas N.A. (2001). Molecular interactions in poly(methacrylic acid)/poly(*N*-isopropyl acrylamide) interpenetrating polymer networks. J. Appl. Polym. Sci..

[15] Lafourcade C., Sobo K., Kieffer-Jaquinod S., Garin J., van der Goot F.G. (2008). Regulation of the V-ATPase along the Endocytic Pathway Occurs through Reversible Subunit Association and Membrane Localization. PLoS ONE.

[16] Ro B.I., Dawson T.L. (2005). The Role of Sebaceous Gland Activity and Scalp Microfloral Metabolism in the Etiology of Seborrheic Dermatitis and Dandruff. J. Investig. Dermatol. Symp. Proc..

[17] Slavin S., Burns J., Haddleton D.M., Becer C.R. (2011). Synthesis of glycopolymers via click reactions. Eur. Polym. J..

[18] Sunasee R., Narain R. (2013). Glycopolymers and Glyco-nanoparticles in Biomolecular Recognition Processes and Vaccine Development. Macromol. Biosci..

[19] Ahmed M., Wattanaarsakit P., Narain R. (2013). Recent advances in the preparation of glycopolymer bioconjugates. Eur. Polym. J..

[20] Miura Y., Hoshino Y., Seto H. (2016). Glycopolymer nanobiotechnology. Chem. Rev..

[21] Dan K., Ghosh S. (2012). PH-responsive aggregation of amphiphilic glyco-homopolymer. Macromol. Rapid Commun..

[22] Wang Y., Zhang X., Han Y., Cheng C., Li C. (2012). pH- and glucose-sensitive glycopolymer nanoparticles based on phenylboronic acid for triggered release of insulin. Carbohydr. Polym..

[23] Guo W., Wang T., Tang X., Zhang Q., Yu F., Pei M. (2014). Triple stimuli-responsive amphiphilic glycopolymer. J. Polym. Sci. Part A Polym. Chem..

[24] Yilmaz G., Guler E., Geyik C., Demir B., Ozkan M., Odaci Demirkol D., Ozcelik S., Timur S., Remzi Becer C. (2018). PH responsive glycopolymer nanoparticles for targeted delivery of anti-cancer drugs. Mol. Syst. Des. Eng..

[25] Tanaka T. (2016). Protecting-Group-Free Synthesis of Glycomonomers and Glycopolymers from Free Saccharides. Trends Glycosci. Glycotechnol..

[26] Tanaka T., Ishitani H., Miura Y., Oishi K., Takahashi T., Suzuki T., Shoda S.I., Kimura Y. (2014). Protecting-group-free synthesis of glycopolymers bearing sialyloligosaccharide and their high binding with the influenza virus. ACS Macro Lett..

[27] Tanaka T., Zhou Y., Tamoto C., Kurebayashi Y., Takahashi T., Suzuki T. (2017). An α2,3-Linked Sialylglycopolymer as a Multivalent Glycoligand against Avian and Human Influenza Viruses. J. Appl. Glycosci..

[28] Tanaka T., Nagai H., Noguchi M., Kobayashi A., Shoda S. (2009). One-step conversion of unprotected sugars to β-glycosyl azides using 2-chloroimidazolinium salt in aqueous solution. Chem. Commun..

[29] Shoda S. (2017). Development of chemical and chemo-enzymatic glycosylations. Proc. Japan Acad. Ser. B Phys. Biol. Sci..

[30] Kolb H.C., Finn M.G., Sharpless K.B. (2001). Click chemistry: Diverse chemical function from a few good reactions. Angew. Chemie Int. Ed..

[31] Hales M., Barner-Kowollik C., Davis T.P., Stenzel M.H. (2004). Shell-cross-linked vesicles synthesized from block copolymers of poly(d,l-lactide) and poly(*N*-isopropyl acrylamide) as thermoresponsive nanocontainers. Langmuir.

[32] von der Ehe C., Czaplewska J.A., Gottschaldt M., Schubert U.S. (2013). Synthesis of thermoresponsive glycopolymers via ATRP of *N*-isopropylacrylamide and *N*-allylacrylamide and subsequent thiol–ene reaction. Eur. Polym. J..

[33] Tanaka T., Okamoto M. (2018). Reversible temperature-responsive and lectin-recognizing glycosylated block copolymers synthesized by RAFT polymerization. Polym. J..

