# Supplementary material for: Synthesis and Aggregation Behavior of Temperature- and pH-Responsive Glycopolymers as Sugar-Displaying Conjugates
Source: Polymers (Basel). 2020 Apr 20;12(4):956. doi: 10.3390/polym12040956 (PMC7240394; doi:10.3390/polym12040956)
Supplement: Supplementary file 1 [file polymers-12-00956-s001.pdf]

Supplementary Information

Synthesis and Aggregation Behavior of Temperature- and pH-responsive Glycopolymers as Sugar-displaying Conjugates

Sotaro Tsuji, Tomohiro Aoki, Shunsuke Ushio, and Tomonari Tanaka*

Department of Biobased Materials Science, Graduate School of Science and Technology, Kyoto Institute of Technology, Matsugasaki, Sakyo-ku, Kyoto 606-8585, Japan.

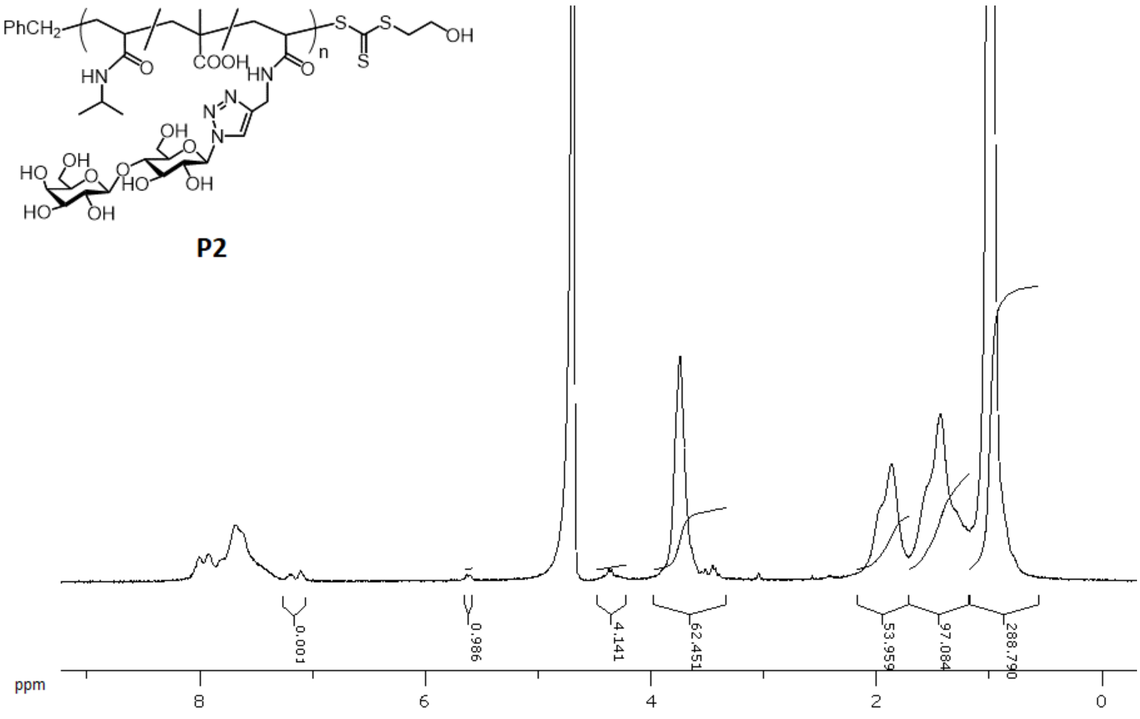

Figure S1 ^{1}H NMR spectrum of **P2** in D_2O.

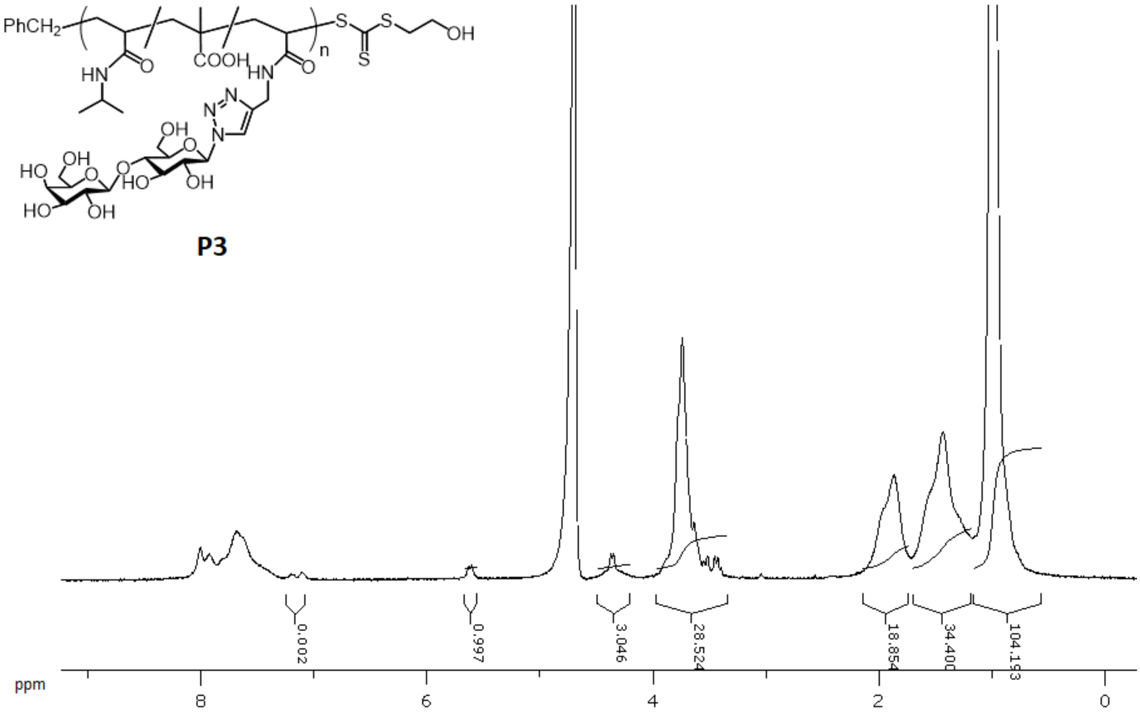

Figure S2 ^{1}H NMR spectrum of **P3** in D_2O.
